# Exploring the Neural Processes behind Narrative Engagement: An EEG Study

**DOI:** 10.1523/ENEURO.0484-22.2023

**Published:** 2023-07-14

**Authors:** Hossein Dini, Aline Simonetti, Luis Emilio Bruni

**Affiliations:** 1 The Augmented Cognition Lab, Aalborg University, Copenhagen 2450, Denmark; 2 Department of Marketing and Market Research, University of Valencia, Valencia 46022, Spain

**Keywords:** dramatic arc, dynamic functional connectivity, EEG, engagement, intersubject correlation, narrative cognition

## Abstract

Past cognitive neuroscience studies using naturalistic stimuli have considered narratives holistically and focused on cognitive processes. In this study, we incorporated the narrative structure, the dramatic arc, as an object of investigation, to examine how engagement levels fluctuate across a narrative-aligned dramatic arc. We explored the possibility of predicting self-reported engagement ratings from neural activity and investigated the idiosyncratic effects of each phase of the dramatic arc on brain responses as well as the relationship between engagement and brain responses. We presented a movie excerpt following the six-phase narrative arc structure to female and male participants while collecting EEG signals. We then asked this group of participants to recall the excerpt, another group to segment the video based on the dramatic arc model, and a third to rate their engagement levels while watching the movie. The results showed that the self-reported engagement ratings followed the pattern of the narrative dramatic arc. Moreover, while EEG amplitude could not predict group-averaged engagement ratings, other features comprising dynamic intersubject correlation (dISC), including certain frequency bands, dynamic functional connectivity patterns and graph features were able to achieve this. Furthermore, neural activity in the last two phases of the dramatic arc significantly predicted engagement patterns. This study is the first to explore the cognitive processes behind the dramatic arc and its phases. By demonstrating how neural activity predicts self-reported engagement, which itself aligns with the narrative structure, this study provides insights on the interrelationships between narrative structure, neural responses, and viewer engagement.

## Significance Statement

Dramatic narratives follow a complex structure termed as the narrative arc. Here, we addressed the complexity of this structure to explore brain responses during narrative cognition. We examined the link between the narrative arc and its six phases with self-reported engagement, and whether brain responses elicited by a narrative can predict engagement levels. Our results showed that the group-averaged engagement ratings followed the dramatic arc model. EEG features predicted group-averaged engagement patterns and also engagement levels in the last two phases. This is the first study to characterize the narrative dramatic arc phases at the neural level. It contributes to the fields of cognitive narratology and neuroscience by extending current knowledge on how the brain responds to narratives.

## Introduction

**Figure 1.**
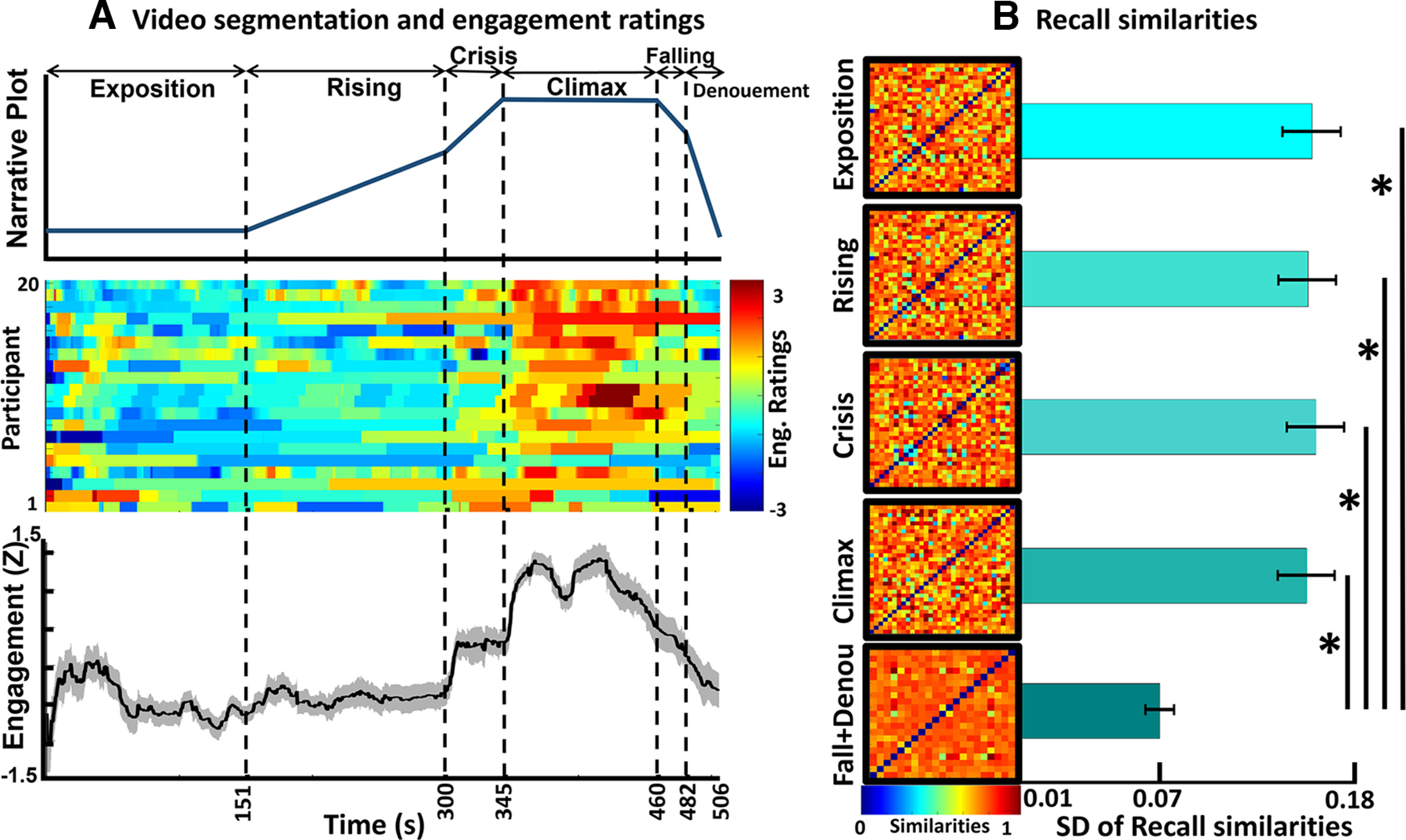
Analysis of event segmentation, self-report, and recall. ***A***, Upper panel, The starting point of each phase as identified by the independent participants after participating in a workshop, including the span of each phase and the phase’s names. The *y*-axis of this panel indicates the expected engagement evoked by the narrative plot. ***A***, Middle panel, The raters’ z-normalized engagement ratings. The hot colors denote higher scores, and the cold colors indicate lower scores. ***A***, Bottom panel, The z-transformed group-averaged engagement ratings. The dashed vertical lines represent the start of the new phase and the end of the previous phase. ***B***, The participants’ recall similarity for each separate phase. In this case, the falling action and denouement phases were combined. The matrices show the similarities between the subjects, with the hot colors indicating higher similarity scores. The horizontal bars show the SDs of the subjects’ recall similarities. The stars indicate that there was a significant difference (*p* < 0.001) between the SDs of the last phase as compared with other phases.

Narratives are naturally engaging stimuli and a useful instrument for understanding affective and cognitive processes (Sonkusare et al., 2019). Regardless of their format (e.g., pictorial, written, audiovisual) or whether they are fictional or real, narratives share a general structure: beginning, middle, and end (Aristotle, 1984; Varotsis, 2018). Since Freytag’s seminal work (Freytag and MacEwan, 1895), it has been theorized that dramatic narratives follow a complex structure known as the narrative dramatic arc. A modern version of Freytag’s arc comprises six phases (Fig. 1*A*, top panel): exposition, rising action, crisis, climax, falling action, and denouement (Laurel, 1991).

A narrative arc intends to create and build tension from the beginning of the story until its climax and then reduce the tension until the end of the narrative (Boyd et al., 2020). Thus, the dramatic arc provides a framework for structuring a compelling and engaging narrative that captures the audience’s attention and keeps them engaged throughout the story (Busselle and Bilandzic, 2009; Bilandzic et al., 2019). However, one thing is the dramatic arc from the author’s point of view, and another thing is its actual effect on the audience. The correspondence of the dramatic arc with the curve of engagement (Song et al., 2021a). could be considered an indication of success in its implementation. Theoretically, many things can influence this correspondence or lack thereof, for instance poor development or representation of the story, the level of familiarity with the story or with its archetypical model/genre, etc. Considering this, self-reported continuous engagement levels provide evidence of the success in the narrative implementation, which can further guide our correlation with brain responses.

Previous neuroimaging studies have provided insights into the relationship between narrative and cognitive processes. They have investigated how the brain responds to narrative event boundaries (Baldassano et al., 2017; Silva et al., 2019; Zheng et al., 2022), movie features (van der Meer et al., 2020), narrative comprehension (Nguyen et al., 2019; Betzel et al., 2020; Song et al., 2021b) and perspective taking (Lahnakoski et al., 2014), among other topics. Particularly, past studies have investigated the relationship between narrative engagement and neural activity. They found that functional connectivity varies depending on perceived narrative transportation (Vaccaro et al., 2021), and potentially predicts fluctuations in attention (Song and Rosenberg, 2021) and engagement levels (Song et al., 2021a). Moreover, specific neural patterns emerge during movie watching and reflect self-reported engagement with the movie (van der Meer et al., 2020), and graph theoretical features are reported to have a relationship with attention (Hong et al., 2013) or emotional moments of audiovisual stimuli (Gupta and Falk, 2015; Vorwerk et al., 2019; Zhang and Liu, 2021). Furthermore, intersubject correlation was shown to capture narrative engagement. Movies evoking emotional responses, which is related to engagement, lead to high levels of brain synchronization and spectral synchronization across individuals (Maffei, 2020). In fact, several studies demonstrated that engaging moments in audiovisual narratives increase spectators’ shared neuronal responses (Dmochowski et al., 2012; Cohen et al., 2017; Poulsen et al., 2017; Song et al., 2021a; Grady et al., 2022). Although these studies used narratives as stimuli, they investigated the connection between engagement and neural responses without focusing on narrative structure. Therefore, the relationship between narrative engagement, brain responses, and narrative arc is underexplored.

Past cognitive neuroscience studies have considered narratives holistically. Hence, whether engagement levels and brain responses relate to each other in each phase of the narrative arc has not yet been investigated. The existence of a defined structure in narratives may reflect an evolutionary need for effective information sharing between individuals (Boyd et al., 2020), but an unanswered question is whether each narrative phase evokes a different brain response. The answer to this question would advance our understanding of the structure and function of narratives from a neurologic perspective. In fact, the “nexus of narrative and mind” (Herman, 2009; p 30) has been the concern of cognitive narratology for the past two decades. While acknowledging the challenges of exploring the field of narrative cognition (Bruni et al., 2021), this study investigates: (1) how the engagement levels fluctuate across a dramatic arc in line with a narrative; (2) whether it is possible to predict this engagement from neural activity; and (3) to further examine narrative comprehension, the idiosyncratic effects of each phase of the dramatic arc on brain responses as well as the relation between engagement and brain responses are explored. We use an explorative approach to fulfill these goals. We presented an excerpt of a movie that followed the narrative arc structure to a group of participants while collecting EEG brain signals. We then asked this group of participants to recall the excerpt, another group to segment the video based on the narrative arc model, and the last group to rate their engagement levels while watching the movie. Using these datasets, we extracted several EEG features to explore which features better represented the link between engagement levels and brain responses in the whole narrative arc and across its phases.

## Materials and Methods

The study comprised four parts with three different groups of participants that used the same stimulus: (1) EEG; (2) recall (freely recalled voice recordings); (3) self-report (self-reported engagement data); and (4) event segmentation (the dramatic-arc phase identification data).

The data collection process for EEG and recall was conducted in November, 2020 as part of a larger study. Participants signed an informed consent sheet before starting the experiment and were paid for their time. The study received approval from the ethics committee for the Technical Faculty of IT and Design at Aalborg University, and it was performed in accordance with the Danish Code of Conduct for Research and the European Code of Conduct for Research Integrity. The codes used in this study are from Song et al. (2021a) and are addressed in Code accessibility part.

### Participants, equipment, and experimental design

#### EEG and recall

Thirty-two right-handed participants (13 females) with M_age_ *=* 26.84 (SD* = *4.33, range: 20–37) wore a 32-channel EEG device (10–20 system; Brain Products). Impedance of the active electrodes was kept below the minimum threshold as stated by the manufacturer (i.e., <25 kΩ) during the whole experiment, and the data were recorded by the Brain Products software at a sampling rate of 1,000 Hz. A virtual reality headset (HTC corporation) placed on top of the EEG device was used for the stimulus presentation. We then told the participants they would see an 8 min 27 s excerpt of an audiovisual movie, and we instructed them to pay close attention to the movie as they would be requested to recall it. The excerpt was from the movie *Pride and Prejudice and Zombies* and can be seen here: https://youtu.be/zp6EPM62wAk. We chose this subplot because it contains the six phases of the narrative arc proposed by Laurel (1991). (The complete arc includes the “inciting incident” phase. However, based on the author’s description and for practical reasons, we merged this phase with the “rising action” phase.) Approximately 50 min after having watched the video, we asked the participants to recall the story from the movie excerpt aloud, and we recorded these recollections with a mobile phone.

#### Self-report

Another group of 20 participants (nine females) with M_age_ *=* 24.80 (SD* = *2.26, range: 22–30) watched the same movie excerpt on a computer. While watching it, they continuously reported their levels of their engagement by adjusting a slider scaled from 1 (“not engaging at all”) to 9 (“completely engaging”). The slider bar was constantly visible at the bottom of the screen during the experiment. This stimulus presentation and recording of responses was controlled by PsychoPy v3.0 software. Before the self-report study, the definition of engagement (inspired by Song et al., 2021a) was provided to the participants.

#### Event segmentation

A third group of 19 participants (eight female) with M_age_ = 26.10 (SD = 2.74, range: 23–32) attended a workshop in which they were introduced to the concept of narrative plots and the six phases of the dramatic arc structure (Fig. 1*A*, top panel). Next, we asked them to watch the movie excerpt and time stamp the moments at which each of the phases started and ended in the movie. In this study, we considered the modern version of Freytag’s arc comprising six phases (Fig. 1*A*, top panel): exposition, rising action, crisis, climax, falling action, and denouement (Laurel, 1991). In the exposition phase, the story’s contextual information is given. In the rising action phase, the tension created by the conflict is escalated and intensified. In the crisis phase, a dilemma related to the conflict occurs. In the climax phase, there is a turning point in which a decision is made, or the dilemma is solved. From a modern perspective, the climax represents the section with the most action. In the falling action phase, the story focuses on secondary conflicts and plots. In the denouement phase, the story comes to an end. A recent study has presented quantitative evidence that narratives follow this general narrative arc (Boyd et al., 2020).

### Data preprocessing

#### EEG dataset

**Figure 2.**
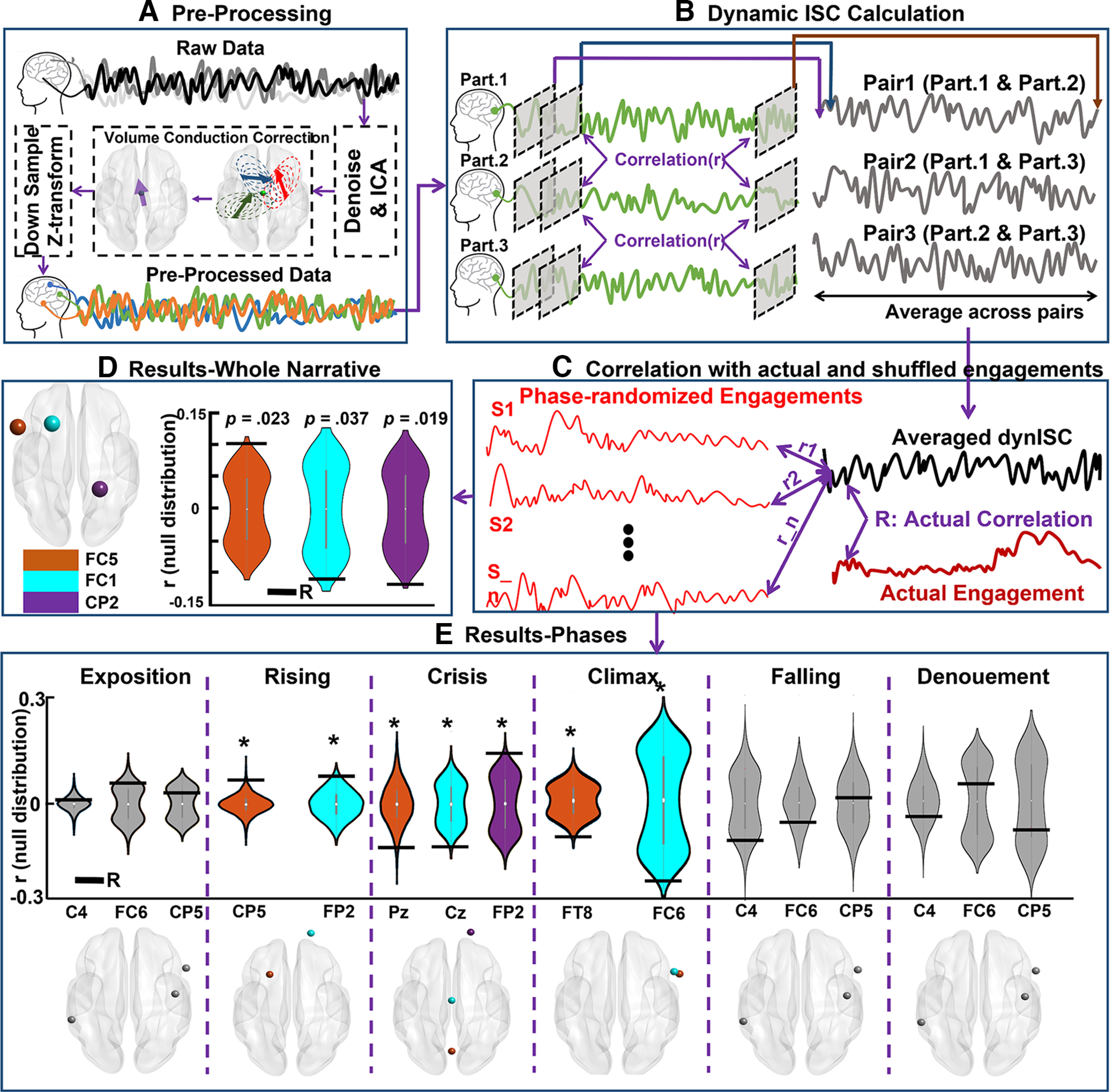
The procedure for calculating the dynamic intersubject correlation (dISC) and the results thereof. ***A***, The steps in preprocessing the EEG data. Each participant’s data went through a preprocessing stage in which the raw data were de-noised. After this, the volume conduction effect was corrected by implementing the current source density approach and then downsampling and z-transforming. ***B***, The steps for calculating the dISC for three participants (from a total of 32) and one channel (out of all the channels). The defined window (indicated by the gray) was applied to every channel for all participants. After this, we calculated the correlation between the participants’ corresponding channels for all participant pairs. Thus, this figure demonstrates the correlations for all three participant pairs as an example. The correlation between each window pair is one sample point of pairwise correlations (shown by the purple, blue, and brown arrows at the top of panel). Sliding the window throughout the entire signal, we obtained a correlation signal for the three participant pairs. The average for these correlation signals was then used in the next step. ***C***, The procedure for the statistical analysis that was conducted using a permutation test. First, we calculated the actual correlation between the averaged dISC and group-averaged engagement rates. Then, we phase-randomized the group-averaged correlations 10,000 times and calculated the correlation between the averaged dISC and each of these phase randomized engagement ratings. Thus, a null distribution and an actual correlation was obtained. ***D***, The results from the comparison of the actual correlation to the null distribution. The orange, cyan, and, purple violin plots correspond to the FC5, FC1, and CP2 channels, respectively. The location and the obtained *p*-values are reported in this panel. The black horizontal lines represent the observed correlations. ***E***, The results from the comparison of the actual correlation to the null distribution, separately for each phase of narrative. The first row shows the actual correlation against the null distribution and the second row indicate the channel locations which their correlation is reported. The phases in which dISC was significantly correlated with engagement ratings (rising, crisis, and climax) are shown with colorful violin plots and channel locations. The phases in which dISC failed to significantly predict the engagement ratings (exposition, falling, and denouement) are plotted in gray. Total of two channels (CP5 and FP2) in rising, three channels (Pz, Cz, and FP2) in crisis, and two channels (FT8 and FC6) in climax were the predictor channels. For the nonsignificant phases (exposition, falling and denouement), we just plotted three examples of the channels (C4, FC6, and CP5) to show where the actual correlation is located against the null distribution.

The EEG signals were first passed through a third-order Butterworth filter that had 1- to 40-Hz cutoff frequencies to remove low and high frequency noises. After this, channels having the activity above the threshold of the mean ± 3 × SD were detected as noisy channels. In addition, all the channels were visually inspected by the first author to detect bad channels, which were removed from the channel list. The average number of rejected channels per participant was 0.93 ± 0.60. One participant was excluded because of having more than four bad channels. To remove eye-related artifacts and other remaining noises, the filtered data (without noisy channels) were fed into an independent component analysis (ICA) algorithm. Using the second-order blind identification method, the source activities (components) were estimated, and eye-related artifacts and other noise sources were detected. The criteria for detecting and removing the noisy components were that the spectral activity, time course trial-by-trial activity, and topo-map of all the components were evaluated by the first author and confirmed by an expert. All the noisy components were removed from the components list. The information regarding the component’s activity was calculated and plotted using the EEG-Lab toolbox. The average number of rejected components per participant was 4.62 ± 0.86. Next, using the calculated ICA coefficients, the data were turned back from source space to channel space, and the de-noised data were obtained. The rejected channels were then interpolated by the spherical spline method using the information from six surrounding channels in the FieldTrip toolbox. Afterward, the de-noised data were re-referenced to the average activity of all the channels. Next, we downsampled the EEG data to 200 Hz by segmenting the EEG signal into bins of averaged data from five sample points (Silva et al., 2019). Since a major part of the current study was based on functional connectivity analysis, an obstacle was the presence of volume conduction, which causes spurious connectivity values among EEG channels (Khadem and Hossein-Zadeh, 2013). These spurious connectivities exist because channels are far from source activities, so the activity of each neural source is picked up by multiple channels. To reduce the volume conduction effect, we used the current source density (CSD) method (Mitzdorf, 1985). This method obtains the spatial properties of each de-noised channel while neglecting the effect of other channels by using the second spatial derivative of the EEG recorded in that channel (Dini et al., 2020). The CSD toolbox (Kayser and Tenke, 2006) was used to apply the CSD method to the de-noised data with the medium spline flexibility of m = 4, as delineated by Dini et al. (2020) and Fitzgibbon et al. (2015). Finally, the data obtained after the implementation of CSD were z-normalized across time and downsampled to 200 Hz to reduce the calculation load. This preprocessing procedure is demonstrated in Figure 2*A*.

### Statistical analyses, data processing

In this section, we detail the methods and the implemented statistics. To facilitate easy reading, we have provided the results of self-report and event segmentation here together with the methodology employed to obtain them.

#### Event segmentation

As previously mentioned, 19 participants attended a workshop in which they were asked to indicate the times at which each phase of the dramatic arc started after they were provided with the definitions for each phase. Using the indicated times, we explored the moments that participants consistently mentioned as the start of each phase. Implementing Silva et al. (2019) method, the number of participants’ indications that were different from the chance level was calculated using 3 s as a window of coincidence (Baldassano et al., 2017). Shuffling the number of observations 1000 times, a null distribution was generated. Then, the coinciding time points with a significance threshold of *p < *0.05 were tested. At least eight of the participants (42.11%) should have coinciding answers that could not be explained by chance (for more on choosing the starting time points, see Silva et al., 2019). This approach resulted in the data detailed in Table 1. The dramatic arc presented by the participants is illustrated in Figure 1*A*, upper panel.

**Table 1:** The starting time points indicated by the participants

Phase	Starting point (s)	Duration (s)	Number of participants who agreed (%)	SD of all responses (s)
Exposition	0	151	19 (100%)	0
Rising action	151	149	13 (68.42%)	95.83
Crisis	300	45	10 (52.63%)	65.92
Climax	345	115	12 (63.16%)	32.37
Falling action	460	22	17 (89.47%)	23.74
Denouement	482	24	18 (94.74%)	4.05

This table shows the time stamps for starting and duration made by independent raters. The choice of the starting time point of each phase of the dramatic arc was obtained from the data of 19 participants that indicated these moments. From the starting time points, the duration of each phase was calculated. The number of participants that indicated the same time points statistically and the standard deviation (SD) of all responses are indicated in the last two columns, respectively.

#### Self-report

We then evaluated how engagement patterns changed across time as the participants experienced the story. All the participants’ button presses were recorded and re-sampled to 200 Hz to match them with the EEG data for further correlation analysis. To match the engagement scores with the EEG data, we first defined a null engagement matrix with a size equal to the EEG length, having 200 sample points per second. Then we replaced the null values with the values referring to the engagement scores recorded by the Psychopy software in each time point. Continuous engagement ratings that were the same length as the video were obtained and then z-normalized across time. The participants’ engagement ratings are demonstrated in Figure 1*A*, middle panel. After this, we analyzed whether the engagement patterns were synchronized across the subjects. To do so, we calculated the pairwise correlation across engagement ratings using the ratings of all the possible participant pairs (Song et al., 2021a). The results indicate that there was a significant positive correlation between pairwise engagement ratings (mean Pearson’s *r*_(18)_ *=* 0.40 ± 0.20). We calculated the average r by z-transforming all pairwise Pearson’s correlation *r* values to z space using Fisher’s method, calculating the average z values, and then transforming the averaged z value back to *r* value. The correlations were significantly positive in 89.47% of the pairwise correlations (false discovery rates were corrected for number of statistical tests; corrected *p < *0.05). Since the participants’ engagement ratings were significantly correlated, the group-averaged engagement rating was considered representative of stimulus-related engagement (Fig. 1*A*, bottom panel). A qualitative evaluation of the group-averaged engagements revealed that this engagement followed the dramatic arc pattern (e.g., engagement peaks in the climax phase and gradually decreases in the last phases).

#### EEG

##### Dynamic intersubject correlation

To investigate whether neural brain activity patterns were modulated by engagement, we used dynamic intersubject correlation (dISC). ISC is a data-driven method that correlates individuals’ neural data to others’ neural data to test whether participants perceive the same stimulus in similar ways and at the same time while neglecting subjective differences (Nastase et al., 2019). ISC is an approach that is well suited to both EEG and fMRI datasets as it captures the stimulus-driven patterns of neural activity (Simony et al., 2016; Petroni et al., 2018; Finn et al., 2020; Imhof et al., 2020; Dini et al., 2023). Moreover, to identify the brain regions’ patterns that are modulated by stimuli, ISC is considered an alternative method that overcomes the problems of traditional methods (e.g., general linear model) as it reduces intrinsic noises (Simony et al., 2016; Song et al., 2021a). Compared with traditional methods, ISC does not require stimulus repetition and fixed experimental manipulation, which leads to a more naturalistic experimental design (Nastase et al., 2019; Dini et al., 2023). To test the hypothesis that ISC is modulated by engagement (i.e., ISC is higher in the more engaging moments), a tapered sliding window with 15 samples (70 ms) was obtained by convolving a rectangular window with a Gaussian (σ = 3). The window size was chosen based on studies that suggest that the optimal window size for dynamic connectivity analysis is 0.05–0.07 multiplied by the sampling rate (Dini et al., 2020, 2021; Sendi et al., 2021a,b,c, 2022a,b). Then, the designed window was slided on the preprocessed EEG signals to cover the entire duration of the signal, with the step size of 5 ms. Next, within each window, we calculated the Fisher’s z-transformed Pearson’s correlation across corresponding participant channels (e.g., correlation between the first participant’s first channel and the second participant’s first channel) and obtained the ISC for all participant pairs. Repeating the same procedure for the entire signal, we obtained the dISC for all subject pairs. The dISC calculation procedure is demonstrated in Figure 2*B*. To test whether dISC was modulated by engagement, we averaged the participants’ calculated dISC according to the EEG channels. Then, we calculated the correlation of each channel’s dISC with the group-averaged engagement ratings (see the self-report section above) and obtained the actual correlation. To test the statistical significance of the calculated correlation, we used a permutation test. To generate the null distribution, the self-reported engagement ratings were phase-randomized, and the correlation between the calculated dISC and phase-randomized engagements were calculated for each iteration. This null distribution was generated because the phase randomization retained the characteristics of the temporal dynamic, such as frequency and amplitude, but it changed the phase (Song et al., 2021a). Repeating this procedure 1000 times, we obtained a null distribution and tested the significance of the actual correlation assuming a one-tailed significance test with *p =* (1 + number of null *r* values ≥ empirical *r*)/(1 + number of permutations), *R*^2^, and the mean squared error (MSE; find more details in Song et al., 2021a). Figure 2*C* demonstrates the permutation test procedure. All the reported *p*-values are corrected using Benjamini–Hochberg false discovery rate (FDR) correction to account for multiple comparisons (Benjamini and Hochberg, 1995).

##### EEG amplitude

**Figure 3.**
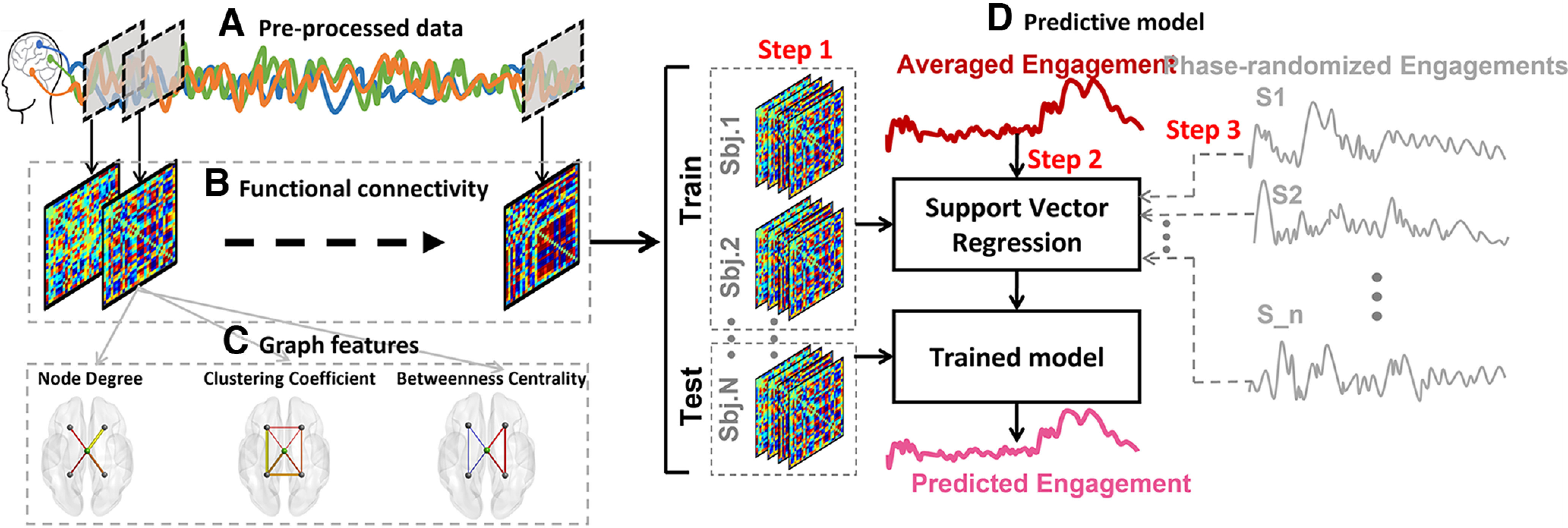
The schematic representation of the dynamic predictive model. ***A***, The sample of preprocessed signals and sliding window (gray squares). ***B***, The functional connectivity among all channels was obtained from the signals occurred within each window. ***C***, Out of each functional connectivity matrix, three graph features, node degree, clustering coefficient, and betweenness centrality, were extracted. ***D***, The procedure of the predictive model. In “Step 1,” we concatenated all the functional connectivity matrices (or graph features) of all subjects and divided it to a train and test sets (the leave-one-subject-out procedure). In “Step 2,” we trained a support vector regression model using the functional connectivities of all subjects but one to predict the averaged engagements and tested the model on the leaved-out subject. The output of Step 2 is the predicted engagement rating. By calculating the correlation between actual engagement and predicted engagement, we obtained the actual correlation. In “Step 3,” we shuffled the engagement ratings as presented in Figure 2 and repeated Step 2 to predict these shuffled ratings. By calculating the correlation between the predicted engagement and each shuffled engagement scores and repeating it for all the 1000 shuffled engagement scores, we obtained a null distribution of correlations. By testing the actual correlation on this null distribution, we obtained the significancy level of the prediction. Note that we implemented this procedure for functional connectivity features and all three graph features separately.

Changes in cognitive and attentional patterns during task performance can be predicted from multivariate fMRI activity (Debettencourt et al., 2015; Haynes, 2015). Although the data used in previous studies are mainly obtained from fMRI, it was worthy to test whether it is possible to predict the engagement patterns using preprocessed EEG amplitudes without extracting any features. To do this, we applied a window with the same characteristics as above and placed the signal, and we then calculated the average EEG amplitude within each window. To predict the engagement ratings with an EEG signal, a leave-one-subject-out (LOO) cross-validation method was implemented using nonlinear support vector regression (SVR) models. The SVR models were trained using the participant’s EEG activity in each window, with one participant’s data being excluded. The SVR models were tested on the held-out participant’s EEG activity in the corresponding window to predict the group-averaged engagement ratings (see above, Self-report). The LOO procedure is illustrated in Figure 3*D*. In each cross-validation fold, the Fisher’s z-transformed Pearson’s correlation between the observed and predicted engagement ratings was calculated to serve as an indicator of predictive performance. We used this metric to evaluate the model’s performance as the main question of this study is whether the temporal dynamic patterns could be captured by the model rather than whether the model could predict the actual values of group-averaged engagement ratings. To test the statistical significance, we used a permutation test. The null distribution was generated by training and testing the same SVR models using the data from the actual brain patterns to predict phase-randomized group-averaged engagement ratings, which were repeated 1000 times. After this, we calculated the prediction accuracy using the abovementioned correlation analysis and generated the null distribution of performance. Finally, we compared the actual model’s performance to the null distribution, assuming a one-tailed significance test with *p =* (1 + number of null *r* values ≥ empirical *r*)/(1 + number of permutations), *R*^2^, and MSE (see Song et al., 2021a).

##### Dynamic functional connectivity

Changes in statistical dependence among different brain regions (i.e., functional network connectivity) could be a helpful approach to predicting changes in participants’ cognitive and attentional states while performing tasks (Rosenberg et al., 2020; Song and Rosenberg, 2021; Song et al., 2021b). To calculate dynamic functional connectivity (dFC), we used the window with the same characteristics as above and placed it to cover the entire signal (Fig. 3*A*). Then, within each window, we calculated the Fisher’s z-transformed Pearson’s correlation across all the time periods of all channels for a single subject (e.g., the correlation between the first channel of the first subject and the second channel of the same subject). The functional connectivity matrices had a size of 32 × 32 to correspond with the channel numbers. This procedure is delineated in Figure 3*B*. Furthermore, to test whether dFC patterns could be used to predict the engagement ratings, we used the same LOO cross-validation approach as used in the “EEG” investigation. The dynamic of each cell of the 32 × 32 matrices during the time course was considered a feature (32 × 31/2 = 496 features). We trained the SVR models using selected features of all participants but one, and we tested the SVR models on the selected features of the held-out participant. We employed a feature selection step before training the model in each cross-validation fold. The features that significantly correlated with group-averaged engagement ratings (one-sample Student’s *t* test with a significance threshold of *p <* 0.010) were selected as candidate features to train the model (Shen et al., 2017). We evaluated the predictive model’s performance using the same approach as above.

##### Graph features

Graph-theory-based methods are helpful tools for understanding brain functional connectivity architecture (Farahani et al., 2019), and they enable better characterization of the behavior of EEG signals that simple linear methods fail to explain (Ismail and Karwowski, 2020). Therefore, it was worthy to explore how dFC structures are related to engagement prediction. To do so, we calculated three graph features using the connectivity matrices obtained from each window throughout the entire signal (Fig. 3*C*). This approach enabled us to evaluate the changes in functional connectivity structures in the time under investigation. Each electrode was considered a node, and the weighted number of correlations between the nodes were considered edges. The calculated graph features consisted of: (1) a node degree (ND), which was obtained from totaling all the weights connected to a node; (2) a clustering coefficient (CC) of a node, which was determined by averaging the weights between the corresponding node and two other nodes that made a triangle with that node; (3) a betweenness centrality (BC) of a node, which was identified by calculating the number of shortest paths in the network that includes the corresponding node. Each of the graph features was calculated at the electrode level, resulting in a matrix with the size of 1 × 32. The detailed definitions of the graph features selected in this study can be seen in García-Prieto et al. (2017). We extracted all the graph features using FastFC toolbox (https://github.com/juangpc/FastFC).

##### Association of dynamic intersubject correlation with frequency bands

Previous studies have investigated how the intersubject correlation is related to the spectral activity of the EEG signals (Dmochowski et al., 2012; Lankinen et al., 2014; Chang et al., 2015; Maffei, 2020). We calculated the frequency information of intersubject correlation inspired by Maffei (2020), using the same window as used in previous parts. (To better understand this part, refer to Fig. 2*B*; however, instead of calculating the correlation of the signals of two windows, we calculated Eq 1.) Within each window, we calculated the dISC spectral activity of all subject pairs. In each subject pair and each window, dISC spectral was defined as the “magnitude-squared coherence of homologous channel activity” using Equation 1 from Maffei (2020):

(1) $$ISC_{i_{AB}}(f)=\frac{|G_{i_{A}i_{B}}(f){|}^{2}}{G_{i_{A}i_{A}}(f)G_{i_{B}i_{B}}(f)}.$$

Where A and B refer to subject A and subject B, i refers to the ith channel, $G_{i_{A}i_{B}}(f)$ refers to the cross-spectral density in channel i of two subjects, and $G_{i_{A}i_{A}}(f)$ and $G_{i_{B}i_{B}}(f)$ represent the auto-spectral density of channel i of subject A and subject B, respectively. We calculated the $ISC_{i_{AB}}(f)$ for all subject pairs and all the channels, resulting in (30 × 29)/2 = 435 subject pairs. By averaging over subject pairs, we obtained the ISC (f). Next, we divided the resulting spectral density into four frequency bands: ISC-δ (1–4 Hz), ISC-θ (4–7 Hz), ISC-α (8–13 Hz), and ISC-β (14–30 Hz). By repeating the same procedure for all the windows, we obtained the dISC-bands.

#### Recall

We manually transcribed the full audio files recorded in recall. We cleaned the transcripts by removing unrelated sentences and words (e.g., “if I remember correctly,” “what did he say,” “hmm,” etc.) for further analysis. We then segmented each participant’s transcribed recalls into six different phases based on the movie moments identified by the group of participants in the event segmentation section. That is, we assigned each sentence to one of the six phases based on whether the events described in the sentence occurred within the phase’s time period. To evaluate the similarities in the participants’ recalls in each phase, we used latent semantic analysis (LSA; Landauer et al., 2013; Nguyen et al., 2019). LSA is a statistical method used to represent text similarity in semantic spaces and that has human-like performance (Landauer et al., 1998). We combined all the preprocessed recalls (text-type documents) from each phase for all participants (32) in a single big document. The LSA method was then used to identify every word in the big document and to remove repetitive and infrequent words. Next, the main components of the big document were extracted by applying a singular value decomposition method. We set the number of components to 20, which was the optimal number of components for each phase with the least perplexity that was suggested by the linear discriminant analysis (LDA) model that searched through 5, 10, 15, and 20 candidate components. As an output, the method decomposed the existing words to the bags of words (i.e., components) with similar meanings. After this, it assigned scores to each participant’s document based on their relationship with the extracted components. Finally, by calculating the pairwise distance (i.e., cosine distance) of the participant’s documents, we obtained the content similarity between all possible pairs contained in participant’s recalls.

### Code accessibility

The raw data collected and used in this study is freely available here: https://zenodo.org/record/7871245#.ZErwxnZBxPY. The code created for the preprocessing, processing, and analyses performed in this study is freely available here: https://github.com/hosseindini/Neural-Processes-behind-Narrative-Engagement. The code and data can be used by other researchers with the condition of citing the present study. To run the code, we used a computer with Windows 10 as the operational system and the following configuration. CPU: AMD Ryzen ThreadRipper 3960 × 3.8 GHz Processor, 24 cores, GPU: RTX 3090, 24GB, Motherboard: ASUS ROG Zenith II Extreme - bundkort - udvidet ATX - Socket sTRX4 - AMD TRX40, and RAM: CORSAIR Vengeance LPX - DDR4 - 64 GB: 4 × 16 GB - DIMM 288-PIN.

## Results

This section presents the results obtained from the methods described in the EEG and recall sections in Materials and Methods.

### Recall

#### Participants recall the information of the falling action and denouement phases in more similar ways than in other phases

As mentioned in Materials and Methods, we segmented the transcribed recalls and assigned each segment to one of the phases. The length of the falling action and denouement phases was short compared with the other phases, so we merged the recall documents of those phases to ensure robust statistics. The averages and SDs for the number of words in each phase are as follows: exposition (M = 25.15, SD = 11.22), rising action (M = 17.19, SD = 8.34), crisis (M = 11.36, SD = 7.26), climax (M = 16.90, SD = 8.15), falling action and denouement (M = 13.45, SD = 8.94). A one-way ANOVA that had the five phases as the independent variable revealed that there was a significant difference in the number of words in the phases (*F*_(4,155)_ = 15.11, *p* < 0.001). To explore which phases differed from the others, we conducted a Dunn–Sidak *post hoc* analysis. The results showed that the significant differences occurred only between the exposition phase and the other phases. This difference between the exposition phase and the other phases is expected as the exposition phase introduces the plot, main characters, and context and, therefore, contains more information than the other phases, which necessitates the explanation of more words Next, we applied LSA to the transcribed recalls of each phase and obtained the between-subjects similarity matrices as well as their SDs (Fig. 1*B*). Following this, we described the mean and SD of the between-subject similarities for each phase: exposition: M = 0.94, SD = 0.15; rising action: M = 0.94, SD = 0.15; crisis: M = 0.94, SD = 0.16; climax: M = 0.94, SD = 0.15; falling action and denouement: M = 0.97, SD = 0.07. As the results suggest, the falling action and denouement phase had the maximum average similarity and the lowest SD among the phases. Since we were interested in exploring how the between-subject similarities changed in each phase, we tested the statistical significance of SDs across the phases. Thus, we implemented the same one-way ANOVA as before but used the SDs obtained from each phase as the dependent variables. The results showed that there was a significant difference among the phases (*F*_(4,45)_* = *2.53, *p = *0.015). Through a Dunn–Sidak *post hoc* analysis, it was revealed that this difference was because of the comparison of the SD of the falling action and denouement phase against all the other phases (*p < *0.05), where this phase’s SD was the lowest among all the phases. The results are illustrated as bars beside the similarity matrices in Figure 1*B*. The greatest recall similarity patterns in the falling action and denouement phase implies that the participants recalled this phase in the same way (i.e., the same information was recalled).

### EEG

#### Cross-subject neural synchrony follows the pattern of the narrative dramatic arc

As discussed in Materials and Methods, we calculated the dISC of the EEG signals, which served as an indicator of subjects’ neural synchrony when exposed to the narrative dramatic arc. After this, we tested our predictions of the group-averaged engagement ratings (self-report) using dISC patterns. We implemented a LOO cross-validation method followed by a permutation test to compare the actual correlation with the null distribution. The results revealed that the dISC significantly predicted the group-averaged engagement ratings (two-tailed nonparametric test, *p < *0.05). The dISCs of three electrodes were significantly correlated with group-averaged engagement ratings: FC5 (*r = *0.11, *p = *0.023), FC1 (*r* = −0.11, *p = *0.034), CP2 (*r* = −0.10, *p = *0.019). Out of these three channels, FC5 was positively correlated with engagement ratings, meaning that cross-subject neural synchrony increased in the more engaging moments of the narrative and decreased in the less engaging moments of the narrative. FC1 and CP2 were negatively correlated with engagement ratings, meaning that the cross-subject neural synchrony decreased in the narrative’s more engaging moments and increased in the narrative’s less engaging moments. The event segmentation results and the engagement ratings indicate that the most engaging moment of the excerpt was contained within the climax phase, whereas the less engaging moments coincided with the other phases. FC5 showed the highest synchrony among the three significantly correlated channels. Two of these channels were concentrated in the frontal-central lobe of the left hemisphere (FC5 and FC1). Therefore, the results suggest that the dISC follows the narrative dramatic arc pattern in the regions concentrated in the left hemisphere frontal lobe. The results are demonstrated in Figure 2*D*.

#### EEG amplitude did not provide sufficient information to predict group-averaged engagement ratings

**Figure 4.**
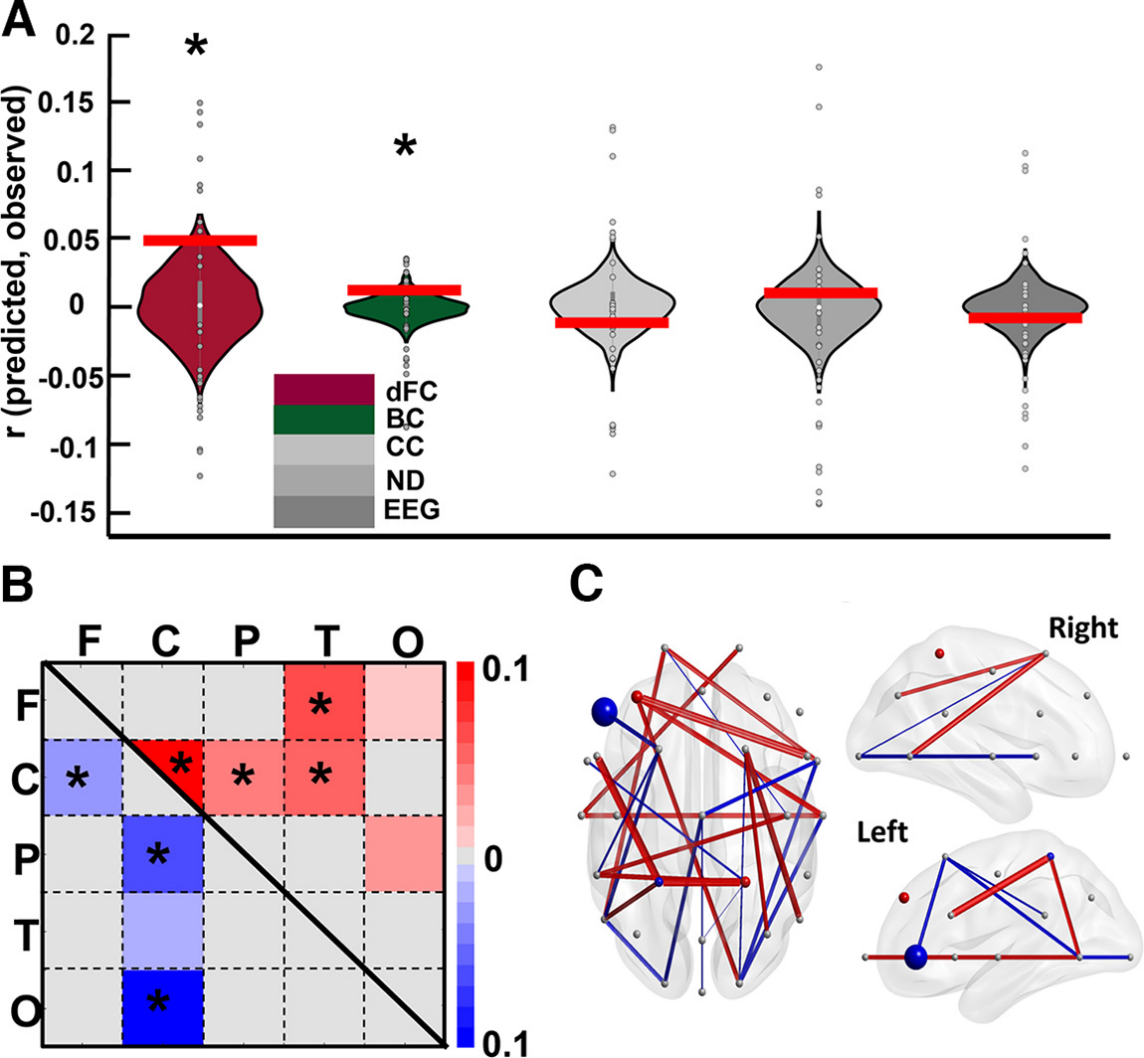
The engagement level predictions for the whole narrative. ***A***, The testing of the actual correlation against the null distribution. Each violin plot refers to the prediction results for each EEG feature. The colored violin plots refer to statistically significant prediction features, and the gray plots refer to the features that were not statistically significant. The dark red violin plot refers to the dFC feature, and dark green plot refers to the BC feature. The light to dark gray spectrum refers to the CC, ND, and EEG features, respectively. The gray circles in the violin plots show the actual correlation obtained in each cross-validation fold with reference to each participant. The red horizontal lines show the observed correlation. The stars indicate that the corresponding feature had been a predictor that was chosen because of it being significantly higher than a chance occurrence. ***B***, The predictor FCs that are either positively (red) or negatively (blue) correlated with engagement ratings across brain regions (F = frontal, C = central, P = parietal, T = temporal, O = occipital). Each cell represents the average number of times that each region played a predictor role. The light to dark colors (red and blue) refer to the strength of their score, which is the number of times they were considered a predictor divided by the length of all possible connections with other channels. To show the positive and negative features simultaneously, we used an upper triangle for positive and a lower triangle for negative features. The original features are symmetrical. ***C***, the predictor FC features and channels on the scalp from three perspectives. The predictor FCs that were averaged in panel B are shown individually here in that the positively correlated items are indicated by red and the negatively correlated items are indicated by blue. The predictor channels obtained from the BC features are represented as colored dots, with bigger dots denoting higher proportions.

We tested whether changes in group-averaged engagement ratings could be predicted using preprocessed EEG amplitudes. The models trained and tested based on EEG amplitudes did not result in significant predictions of group-averaged engagement ratings (*p = *0.521, *r* = −0.015, MSE* = *1.064, *R*^2^ *=* −0.064) in any of the EEG channels. The reported values were the result of a correlation analysis between predicted engagements based on EEG amplitudes and actual group-averaged engagement ratings that were averaged across the channels. Furthermore, we added a feature selection section in every cross-validation iteration to increase the neural features’ specificity. Therefore, only the EEG time courses (channels) that were consistently correlated with the group-averaged engagement ratings (one-sample Student’s *t* test, *p <* −0.010) were fed into the SVR models instead of the data from all channels (Shen et al., 2017). Training the models using the selected features still did not result in robust predictions, and the EEG amplitudes were not significantly correlated with engagement ratings (*p = *0.742, *r* = −0.005, MSE* = *1.032, *R*^2^ *=* −0.032). Therefore, our results show that EEG amplitudes failed to predict the group-averaged engagement ratings. The results obtained from testing the actual prediction values on the generated null distribution is illustrated in Figure 4*A* in the form of a gray violin plot.

#### dFC patterns could predict the group-averaged engagement ratings in which the central region plays a vital role

The models trained and tested using extracted functional connectivity (FC) features successfully predicted the group-averaged engagement ratings (*p = *0.029, *r = *0.049, MSE* = *1.095, *R*^2^ = −0.095), as shown in Figure 4*A* in the form of a dark red violin plot. The null distributions are positively skewed in this figure, which might be because they were generated by training the model to predict the phase-randomized engagements and because the model was tested to predict the same data using the held-out participant’s dFC. Nevertheless, the prediction accuracy was significantly higher than the generated null distribution.

To investigate which brain regions significantly contributed to the predictions of group-averaged engagement, we visualized the FC features that were consistently selected as predictors (see Data processing; EEG; and Dynamic functional connectivity sections) in every cross-validation fold seen in Figure 4*B*,*C*. A total of 264 FC features that were positively correlated with engagement and a total of 136 FC features that were negatively correlated with engagement were both consistently selected. We called these features “predictive FCs.” Each participant had a matrix consisting of these positively and negatively correlated features. Figure 4*C* shows the selected FC features averaged across participants, with red denoting positively correlated features and blue signaling negatively correlated features. To have a better understanding of these predictive FCs, we first defined the five canonical brain regions: frontal (F), central (C), parietal (P), temporal (T), and occipital (O). After this, we totaled the number of predictive FC features included in each region, resulting in a 5 × 5 matrix for each participant. Next, we calculated the proportion of predictive FCs relative to the total number of possible connections among regions to create a proportion matrix for each participant. This was done by dividing the 5 × 5 matrices by the lengths of all possible connections among regions (i.e., networks). Finally, by averaging the proportion matrices across the participants, we obtained the proportion score of all possible connections (see Fig. 4*B*).

To evaluate whether the defined networks, containing predictive FC information, were present more frequently than would be explained by chance, we implemented a one tailed nonparametric test on the number of predictive FCs within and between regions (the significant regions are indicated in Fig. 4*B*, stars; FDR-corrected *p < *0.001). All the *p*-values were then corrected according to the number of test repetitions using the Benjamini-Hochberg method (Benjamini and Hochberg, 1995). Our results suggest that the within-central, between-central, and all other regions except occipital were constantly and significantly selected as predictive FC features. Although there was also a frontal-temporal connection that was able to predict the engagement pattern, our results suggest that the central region plays a vital role in predicting engagement patterns.

#### Graph features of central and frontal regions could significantly predict the group-averaged engagement ratings

We examined whether the graph features extracted from the connectivity matrices described in the previous section could predict engagement ratings. The SVR models were trained and tested using three graph features: ND, CC, and BC. The procedure for the LOO cross-validation and the subsequent permutation test was the same as described previously. The results show that BC significantly predicted the group-averaged engagement ratings (*p = *0.035, *r *= 0.012, MSE* = *1.101, *R*^2^ *=* −0.101). As shown in Figure 4*A* in dark green, the actual correlation is significantly higher than the generated null distribution. However, ND and CC failed to significantly predict the engagement ratings (ND: *p = *0.423, *r = *0.009, MSE* = *1.071, *R*^2^ *=* −0.071; and CC: *p = *0.831, *r* = −0.010, MSE* =* 1.059, *R*^2^ *=* −0.066). As shown in Figure 4*A*, gray, these aspects were not significantly distanced from the null distributions. For BC, we evaluated which channels were consistently selected in each cross-validation fold. The results showed a total of three channels, with the CP2 channel being positively correlated and the CP1 and F7 channels being negatively correlated with the group-averaged engagements. In Figure 4*C*, the predictive channels with positive correlations are labeled in red, and those with negative correlations are labeled in blue. Next, we evaluated whether the repetition of these channels is higher than would be expected by chance by calculating the channels’ proportion of predictive BC relative to the total number of possible channels (as above). The results show that all three channels’ BCs were significantly selected in cross-validation folds (CP2: proportion score = 0.066, corrected *p < *0.001; F7: proportion score = 0.433, corrected *p < *0.001; and CP1: proportion score = 0.033, corrected *p < *0.001). Therefore, the graph theoretical feature from central and frontal regions could significantly predict the group-averaged engagement ratings.

#### dISC-δ, dISC-θ, and dISC-β could predict the group-averaged engagement ratings

**Figure 5.**
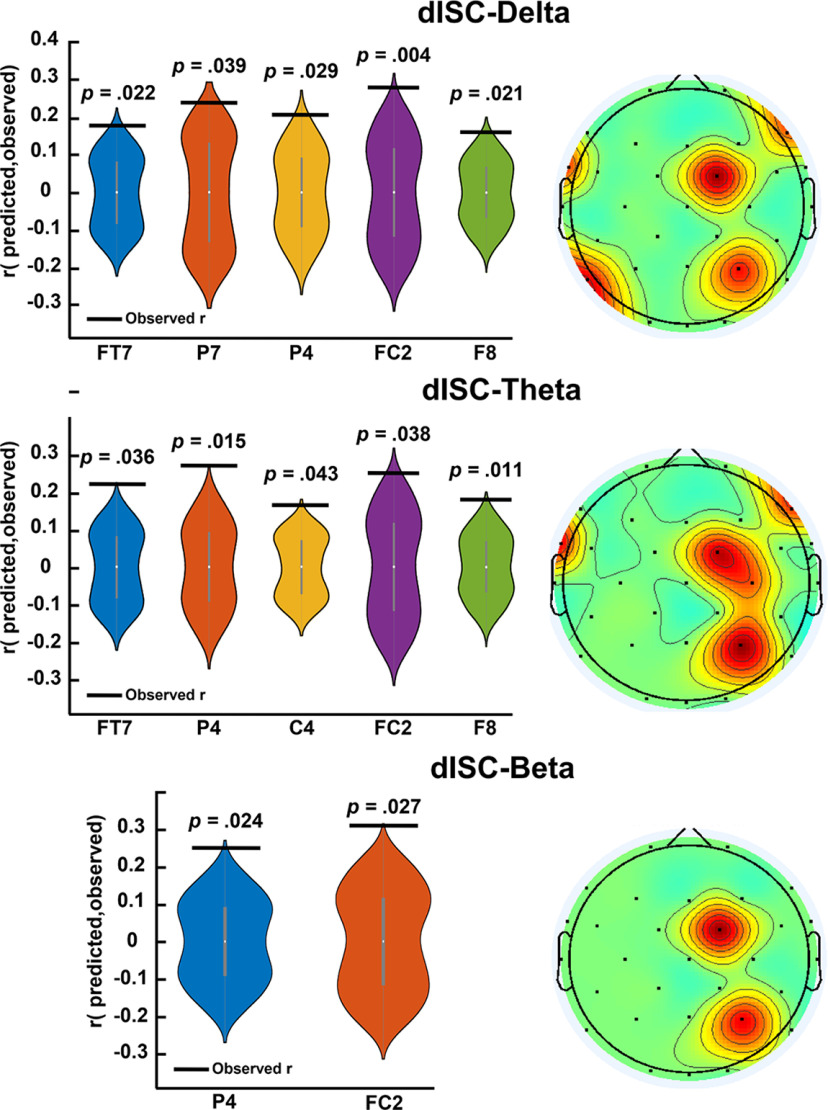
The results for predicting group averaged engagement ratings using dISC-bands. The violin plots refer to the null distribution of the correlation value (*r*) between surrogated engagement scores and the calculated dISC-bands, only for the significant channels. The observed *r*, which is the correlation between actual engagement scores and dISC-bands, is also indicated black lines over violin plots. The topo-plots indicate the observed r of the channels that could significantly predict the engagement scores, on the scalp. The *p*-values are reported above each violin plot, and all of them are corrected. In dISC-δ and dISC-θ, five channels could significantly predict the group-averaged engagement ratings. The spatial activity of the predictor channels can be seen on the topo-plot beside the violin plot. In dISC-β, two channels could significantly predict the engagement scores and the spatial distribution of the predictor channels can be seen in the topo-plot.

To test whether dISC fluctuates as a function of spectral bands, we calculated dISC-bands in δ (dISC-δ), θ (dISC-θ), α (dISC-α), and β (dISC-β) frequency bands. Next, having the correlation between dISC and group-averaged engagement ratings, we tested whether dISC-bands could predict the engagement ratings by implementing a LOO cross-validation method followed by a permutation test. If dISC-bands could predict the engagement ratings, we argue that there was a link between dISC and dISC-bands, since both features could predict the same engagement ratings: one considering the neural synchrony across subjects and the other considering cross-spectral density across subjects. The results showed that dISC-δ, dISC-θ, and dISC-β, but not dISC-α, could significantly predict the engagement ratings. The results concerning the statistically significant predictive channels for each frequency band are as follows (Fig. 5). dISC-δ: FT7 (*r = *0.17, *p = *0.022), P7 (*r = *0.24, *p = *0.039), P4 (*r =* 0.20, *p = *0.029), FC2 (*r = *0.27, *p = *0.004), F8 (*r = *0.16, *p = *0.021). dISC-θ: FT7 (*r = *0.22, *p = *0.036), P4 (*r = *0.27, *p = *0.015), C4 (*r = *0.16, *p = *0.043), FC2 (*r = *0.25, *p =* 0.038), F8 (*r = *0.18, *p = *0.011). dISC-β: P4 (*r = *0.25, *p = *0.024), FC2 (*r = *0.31, *p = *0.027). All *p*-values were corrected according to the number of repetitions. Thus, all these channels were positively correlated with engagement ratings, meaning that cross-subject spectral density in δ, θ, and β bands increased in the more engaging moments of the narrative (e.g., climax) and decreased in the less engaging moments of the narrative (e.g., falling and denouement). In summary, ISC-δ, and ISC-θ in wider regions of frontal, parietal, and temporal regions were significantly synchronized with engagement scores, while this synchronization occurred in central and parietal regions in dISC-β.

#### Shared neural responses could significantly predict engagement in more engaging phases, whereas dFC and BC could predict engagement in less engaging phases of the dramatic arc

**Figure 6.**
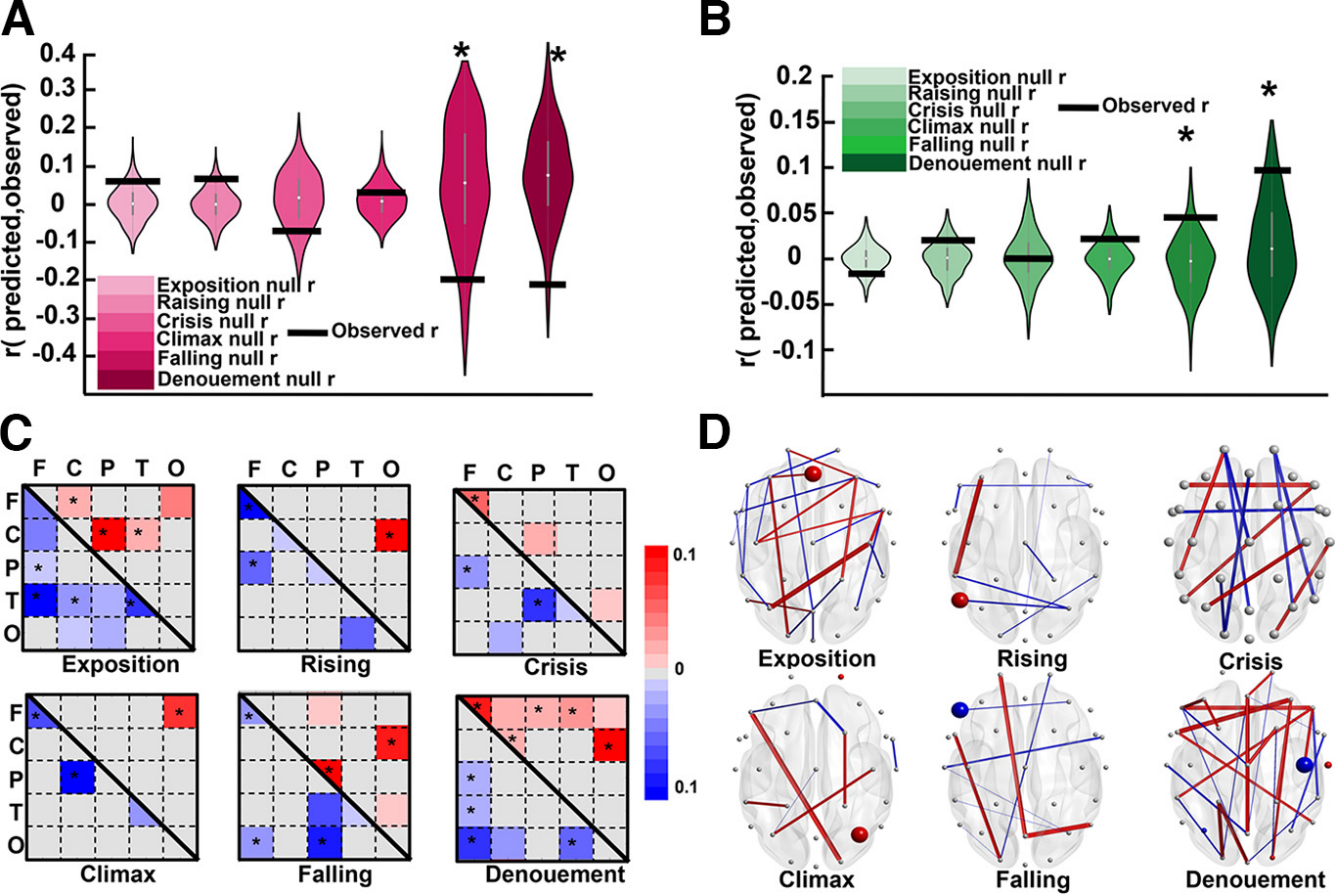
See Figure 4. The predictions of the engagement ratings of each phase using the corresponding features. The black horizontal lines show the observed correlation, and the violins show the null distribution. ***A***, The phase prediction results for all phases using dFC features. It shows that the prediction in the last two phases (falling action and denouement) was significantly higher than could be explained by chance. ***B***, The prediction results using BC features and the significant prediction that occurred in the last two phases. ***C***, The predictor FCs across regions and their proportion scores for each phase. ***D***, The predictive FCs and the channels that the BCs played as predictor roles for each phase.

We made advancements in evaluating neural activity that was stimulated by each narrative phase as opposed to just making predictions for the whole excerpt. For this, we analyzed the possibility of predicting the engagement levels in each phase separately using the data obtained from the brain. We segmented both the group-averaged engagement ratings and neural activity based on the phases delineated in the event segmentation section. We extracted dISC, dFC, and BC features, in the same way that we extracted to predict the whole narrative, this time separately for six phases. After this, we implemented the same approach for predicting dISC and the same LOO cross-validation approach to evaluate whether the feature could predict the engagement ratings. All the methods implemented herein were identical to those explained previously, but the models were fed the neural activity and engagement ratings of each phase, and they were trained and tested separately in this case. For dISC, the results showed that the dISC could significantly predict the engagement ratings for rising, crisis, and climax phases, and failed to significantly predict the exposition, falling, and denouement phases (Fig. 2*E*). In rising, CP5 (*r* = 0.065, *p* = 0.015) and FP2 (*r* = 0.074, *p* = 0.032) were the predictor channels. In crisis Pz (*r* = −0.116, *p* = 0.033), Cz (*r* = −0.113, *p* = 0.011), and FP2 (*r* = 0.135, *p* = 0.014) were the predictor channels. In climax, FT8 (*r* = −0.098, *p *= 0.010) and FC6 (*r* = −0.216, *p* = 0.011) were the predictor channels. Regarding the nonsignificantly predicted phases, we report three samples of channels as representative of all on-significant channels. In exposition: C4 (*r* = 0.011, *p* = 0.416), FC6 (*r* = 0.057, *p* = 0.234), and CP5 (*r* = 0.030, *p *= 0.152). In falling: C4 (*r* = −0.092, *p* = 0.647), FC6 (*r* = −0.054, *p* = 0.379), and CP5 (*r* = 0.015, *p = *0.894), and in denouement: C4 (*r* = −0.044, *p* = 0.733), FC6 (*r* = −0.054, *p* = 0.575), and CP5 (*r *= 0.015, *p = *0.930) are the representative channels. For dFC features, the results show that the models trained and tested using the dFC features of each phase failed to significantly predict the exposition (*p = *0.458, *r = *0.061, MSE* = *1.271, *R*^2^ *=* −0.271), rising action (*p = *0.325, *r = *0.063, MSE* = *1.612, *R*^2^ = −0.612), crisis (*p = *0.627, *r* = −0.072, MSE* = *1.583, *R*^2^ = −0.583), and climax (*p = *0.261, *r* = −0.039, MSE* *=* *1.431, *R*^2^ = −0.431) phases. However, the models significantly predicted the falling action (*p = *0.028, *r* = −0.197, MSE* = *1.798, *R*^2^ *=* −0.798) and denouement (*p = *0.009, *r* = −0.202, MSE* = *1.812, *R*^2^ = −0.812) phases. The results (dFC features) obtained from comparing each phase’s actual correlation with the generated null distribution are demonstrated in Figure 6*A*, which shows that the actual correlation was significantly lower than the null distribution in the falling action and denouement phases. Furthermore, the results from training and testing the models that were fed the BC features of each phase revealed that, for the BC feature, the models failed to significantly predict the exposition (*p = *0.587, *r* = −0.016, MSE* = *1.187, *R*^2^ = −0.187), rising action (*p = *0.145, *r* = −0.020, MSE* =* 1.432, *R*^2^ *=* −0.432), crisis (*p = *0.912, *r = *0.001, MSE* =* 2.722, *R*^2^ *=* −0.722), and climax (*p = *0.178, *r = *0.022, MSE* = *1.647, *R*^2^ = −0.647) phases. However, the models significantly predicted the falling action (*p = *0.051, *r =* 0.045, MSE* = *1.586, *R*^2^ *=* −0.586) and denouement (*p =* 0.041, *r = *0.097, MSE* = *1.387, *R*^2^ *=* −0.387) phases. The results (BC features) from comparing each phase’s actual correlation with the generated null distribution are demonstrated in Figure 6*B*, which shows that the actual correlation was significantly higher than the null distribution in the falling action and denouement phases.

The FCs that were consistently selected as predictors in each cross-validation fold are shown in Figure 6*C*, with a separate label for each phase. The total number of positively and negatively correlated predictor FCs are as follows: exposition: 96 positively and seven negatively correlated; rising action: 0 positively and 170 negatively correlated; crisis: 52 positively and 108 negatively correlated; climax: 14 positively and 130 negatively correlated; falling action: 40 positively and 32 negatively correlated; denouement: 184 positively and 96 negatively correlated. As in the previous section, we examined FCs that acted as predictors in each brain region defined above. Figure 6*C* shows these FCs, with a separate label for each phase. After this, we tested whether these regions were selected in ways that were significantly higher than would be expected for chance selections by using the statistical analysis explained above. The significant predictor FCs are indicated by the stars in the corresponding matrix of each phase in Figure 6*C*. The results demonstrate that, in terms of the phases in which the SVR model could successfully predict the engagement ratings (i.e., falling action and denouement), the frontal lobe and the connection of the frontal region with other regions played a vital role in predicting the engagements: In the falling action phase, the connection was between frontal and occipital, and, in the denouement phase, the connection was between frontal and parietal and temporal regions. Moreover, the central-occipital connection in the falling action phase and the central connection in the denouement phase were also significant predictors. Although the neural activity of the other phases failed to predict the engagement ratings, it is worth mentioning that, in all of them except for the exposition phase, the frontal region was identified as a significant predictor.

We then implemented the approach explained under Materials and Methods, Statistical analyses, data processing, EEG, Graph features section to investigate which BC of which channels were consistently selected as predictors by calculating the proportion scores. In the last two phases, in which the model successfully predicted the engagement ratings using the BC feature, the selected channels, their calculated proportion score, and the *p*-values were as follows: falling action: F7, negatively correlated, proportion score = 0.033, *p = *0.029; denouement: C4, negatively correlated, proportion score = 1, *p < *0.018; P3, negatively correlated, proportion score = 0.066, *p = *0.018; O_2_, positively correlated, proportion score = 0.030, *p = *0.031; T8, positively correlated, proportion score = 0.200, *p *< 0.001. Therefore, in the denouement phase, the highest-scoring channel (C4) was in the central region. Moreover, considering all of the phases, most of the predictor channels were related to the central and frontal regions.

In summary, our results show that the models trained on dFC and BC features were able to significantly predict the engagement ratings of the falling action and denouement phases. Moreover, the frontal and central regions as well as their connection to other regions played an important role in predicting the engagement ratings in the last two phases, which was determined by the evaluation of both dFC and BC features.

## Discussion

A given description possesses narrativity when receivers identify it as being a narrative, regardless of the intentions of the sender in evoking this perception (Ryan, 2005). Higher levels of narrativity are ensured in stories that have a dramatic arc (Ryan, 2007). We addressed the complexity of the dramatic arc to explore brain responses during narrative cognition. Specifically, we examined the link between the phases of a narrative arc and self-reported engagement and whether brain responses elicited by a narrative can predict engagement levels. Our results show that the group-averaged engagements followed the dramatic arc model and could be predicted by dISC, dFC, and BC features. We also predicted engagement in the last two phases by investigating the neural underpinnings of each phase.

The fluctuations in self-reported engagement ratings occurred synchronously for all the participants, reflecting that individuals share states of engagement. Moreover, individuals could identify a narrative arc structure in our stimulus (Fig. 1*A*, upper panel). Notably, we found that perceived engagement at the group level followed the same shape as the narrative arc structure (Fig. 1*A*, bottom panel). The individuals’ synchronous engagement and the shape of its temporal development (i.e., similar to the narrative arc) could be attributed to transportation effects (see Green and Brock, 2000). This finding is also in line with Song et al.’s (2021a) study in which it was shown that emotional moments of a narrative evoke stronger engagement levels. Moreover, previous studies have reported that an individual’s engagement state is evoked by and aligned with tension levels (Busselle and Bilandzic, 2009; Bilandzic et al., 2019). Similarly, our results show that group-averaged engagement increased in the rising action phase, became more pronounced in the crisis phase, and peaked in the climax phase in which there is maximum tension, after which it declines. Therefore, the dramatic arc regulates an individual’s engagement state by fluctuating tension levels.

We then explored whether individuals’ brain activity was modulated by their engagement levels throughout the dramatic arc model. We previously found that different levels of narrativity led to differences in ISC and different levels of perceived engagement, thereby predicting narrativity level (Dini et al., 2023). Cohen et al. (2017) evaluated time perception and its relation to engagement during narrative videos, from which they posited that engagement could be considered subject’s levels of similarity among EEG channels (i.e., ISC). Moreover, the subjective perception of time and neural processing becomes synchronous when watching narratives (Cohen et al., 2017). Dmochowski et al. (2012) stated that extracting correlated components using ISC is related to “emotionally-laden attention.” Thus, ISC might be a marker of engagement (Dmochowski et al., 2012). Correspondingly, our results indicate that the dISC of individuals’ frontal (FC5) electrode was more synchronized in a narrative’s more engaging moments, whereas electrodes also in the frontal (FC1) and central (CP2) were less synchronized. Implementing fully cross-validated models revealed that the dISC of the frontocentral region became more active and involved in predicting moment-by-moment engagement levels (Fig. 6*D*), whereas EEG amplitudes could not make this prediction (Fig. 4*A*). Therefore, the dISC patterns followed the dramatic arc structure. However, while there was an overall positive relationship between dISC and engagement with the narrative for FC5, dISC of FC1 and CP2 showed a negative correlation with engagement ratings. Previous fMRI research also showed that the dISC of certain brain regions has a negative relationship with behavioral engagement of audiovisual narratives (Song et al., 2021a). The pattern considering the average ISC of FC1 and CP2 was organized in a way that subjects’ neural activity was less synchronized in the engaging phases of the dramatic arc (i.e., crisis and climax) and relatively more synchronized in less engaging moments (i.e., exposition, rising action, falling action, and denouement). While previous studies also found a link between engagement and ISC levels (which supports the use of dISC as a marker of engagement), we extended this and argued that the subjects’ neural activity was modulated by the presence of a narrative structure that unfolded as a dramatic arc. This finding is a promising step forward in the understanding of narrative cognition.

We extended our dISC calculation to the frequency domain to explore whether ISC varies as a function of spectral information of the frequency bands. That is, we evaluated whether dISC-bands could significantly predict the engagement ratings. Few studies have investigated the relationship between dISC-bands and engagement; we review this literature and compare them to our findings separately for each frequency band as follows. Regarding the dISC-δ and dISC-θ, Maffei (2020) evaluated the EEG ISC-bands while participants attended to video clips containing different emotional content. They reported that temporal and parietal ISC-δ are modulated by the emotional content of the videos, showing higher synchrony in high arousal, erotic, and fearful emotional content. In addition, they reported that ISC-δ was spread widely over brain regions, where frontal and temporal sites were related to the emotional content of the movies, and posterior sites peaks were related to visual stimulation. In another study, Kauppi (2010) investigated fMRI frequency components of ISC while participants were watching an engaging movie. They found greater ISC in frontal and temporal locations at low frequencies than in high-frequency bands. Chang et al. (2015) reported a strong ISC-δ while participants watched a naturalistic movie clip, and Lankinen et al. (2014) found high ISC at frequencies below 10 Hz while participants watched a silent movie. Another study by Dmochowski et al. (2012) showed that frontal θ power increases during moments of high ISC (a proxy for high engagement) while watching narratives. In line with these studies, our results show that dISC-δ and dISC-θ are significantly correlated with engagement ratings in frontal (F8), temporal (FT7), and parietal (P7 and P4 for dISC-δ, and P4 for dISC-θ) locations. The positive correlation means higher dISC-δ and dISC-θ in more engaging moments (e.g., climax) and lower dISC-δ and dISC-θ in less engaging moments (e.g., falling and denouement). Maffei (2020) found higher ISC-δ in videos containing high arousal content. We speculate that our movie extract also evoked arousal, especially in the climax phase. Dmochowski et al. (2012) found higher frontal dISC-θ in the more engaging moments of the narrative. We had three channels involving the frontal location that were positively correlated with engagement ratings. Furthermore, our results showed that in lower frequencies (i.e., δ and θ), the predictive channels are more spread across the brain compared with the higher frequencies (i.e., α and β), which is in line with the findings of two past studies (Lankinen et al., 2014; Chen and Farivar, 2020). Regarding the dISC-β, Lankinen et al. (2014) investigated the frequency components of ISC while participants attended a silent black-and-white movie excerpt. They reported statistically significant correlations across the brain activity of the subjects in frequencies around 24 Hz frequency, although its source was speculated to derive from the movie framing rate. Dmochowski et al. (2012) showed that the parietal and occipital β powers decreased during moments of high ISC while watching narratives. Our results showed that dISC-β is significantly and positively correlated with engagement ratings in central (FC2) and parietal (P4) regions. This is in line with Lankinen et al. (2014), who reported that ISC-β is mostly correlated across subjects. However, our results do not support the findings of Dmochowski et al. (2012), as they reported a decrease in ISC-β. Regarding the dISC-α, previous studies have reported that ISC-α is modulated by attentional demand, which is a core component of engagement, and the emotional content of movies (Dmochowski et al., 2012; Ki et al., 2016; Maffei, 2020). Contrary to it, our results did not show any significant correlation between dISC-α and engagement ratings. This finding might be explained by the weak modulation of the α band with attentional states compared with ISC (Ki et al., 2016), and that dISC-α is weaker than dISC of other frequency bands during movie watching (Lankinen et al., 2014; Chang et al., 2015). Importantly, the topographical patterns of ISC-bands differ according to the emotional state induced by the stimulus (Maffei, 2020). Therefore, our results might be specific to our stimuli, and modulation of dISC-α with engagement would need more investigations in further studies. In summary, our results showed that δ, θ, and β bands could significantly predict the engagement ratings, suggesting that dISC varies as a function of these frequencies. This finding contributes to unfolding the neural processes behind narrative cognition.

Previous studies have also characterized engagement as well as emotional and focused attentional states (Schreiner et al., 2022), which is closely related to engagement (Busselle and Bilandzic, 2009; Dmochowski et al., 2012) using FC. In a dFC study, Song et al. (2021a) evaluated narrative engagement, sustained attention, and event memory during narrative exposure. They reported that models based on dynamic brain connections could predict engagement states in two independent datasets. Moreover, the default mode network activity, which fluctuated in line with engagement states, was also able to predict sustained attention and recall of the narrative events. In the current study, we explored whether there was a link between engagement states and FC and FC-related features (i.e., BC graph). By using similar cross-validation models, both dFC features and the BC graph feature were significantly correlated with engagement states (Fig. 4*A*). The FC networks’ patterns that acted as predictors revealed that the central and frontal lobe played a vital role in predicting engagement states (Fig. 4*B*). Most of the FC features were positively correlated with engagement, however other FC features showed a negative correlation with engagement ratings. Overall, the positive FCs dominated the negative FCs, resulting in an averaged positive relationship between dFC and engagement. Song et al. (2021a) found that connections within the frontoparietal control network had a negative relationship with engagement. In our study, the negative connections were between frontal and central electrodes and between central and parietal electrodes. Moreover, the predictor graph channels were concentrated on central (CP2, positively correlated; CP1, negatively correlated) and frontal (F7, negatively correlated) channels. BC had an overall positive correlation with engagement levels. In line with the findings on dISC predictors, the dFC confirmed that the neural connections across central and frontal regions were more synchronous with engagement patterns (i.e., more neural activity in more engaging moments of the narrative and vice versa). In addition, the BC graph feature, in line with the dISC (specifically in the CP2 channel), revealed that the central and frontal channels played a crucial role in predicting engagement patterns. The BC graph features have been considered putative hubs in a network (Bullmore and Sporns, 2009). In summary, the analysis of the dFC and BC features showed that predictive channels in frontal and central regions followed the dramatic arc pattern (confirmed by the dISC), either in the same or opposite directions, and that the information flow passed (or possibly accumulated) through the regions of those channels to connect to other brain regions. Hence, these features were important for characterizing predictive brain regions for cognitive, attentional, and engagement states, in addition to ISC.

We have used three main neural measures, dISC, dFC, and graph theoretical features, to explore the relationship between brain activity and perceived engagement to an audiovisual narrative. ISC measures shared responses across individuals receiving the same stimulus, and it is sensitive to engagement levels and shared understanding (Dmochowski et al., 2012; Nguyen et al., 2019). FC assesses coactivations between brain regions (in our case, between electrodes). Importantly, FCs may be particular to a given narrative, as previous research suggests that patterns of connectivity are content-specific (Vaccaro et al., 2021). Graph theoretical analysis considers the nodes and edges of a network to derive network-related features (e.g., connection patterns, their strength, small-worldness; Yamamoto et al., 2022). The three neural measures could predict perceived engagement with the whole narrative through different perspectives: high similarity in neural responses (ISC), interactions between certain brain regions at the electrode level (FC), and specific electrodes playing the role of hubs in the network (i.e., node; BC). Therefore, the results support previous research showing a relationship between ISC and narrative engagement (Dmochowski et al., 2012; Cohen et al., 2017; Poulsen et al., 2017; Maffei, 2020; Song et al., 2021a; Grady et al., 2022) and between FCs and narrative engagement (Song and Rosenberg, 2021; Song et al., 2021a; Vaccaro et al., 2021). In addition, studies characterizing brain networks involved in audiovisual stimuli processing through graph analysis demonstrated the relationship between several graph features and attention (Hong et al., 2013) or emotional moments (Gupta and Falk, 2015; Vorwerk et al., 2019; Zhang and Liu, 2021). Furthermore, the three measurements revealed the prominent role of the frontal and central regions in predicting engagement with a narrative that follows the dramatic arc structure. The three measurements were also able to predict engagement with the narrative at the narrative phase level. While dISC was successful in predicting narrative moments eliciting higher engagement and low shared understanding, FC and BC could predict moments eliciting lower engagement and higher shared understanding. In summary, our results suggest the three metrics complement each other in the assessment of narrative cognition focused on engagement levels.

In addition, the results of engagement prediction in the different phases point to an interesting finding. The dISC feature predicted the engagement ratings of the highly engaging phases of the narrative arc (i.e., climax and crisis). These two phases were characterized not only by high engagement scores but also by low dISC values and high SD of recall similarity. This implies a low shared understanding of these two phases across participants. In summary, in line with other studies, our results from the whole narrative indicate that dISC is predictive of engagement. Specifically, in our study, this prediction happened in the narrative phases where shared understanding is low. In contrast, the dISC failed to predict the engagement scores of the last two phases (i.e., falling action and denouement), where engagement levels were low and shared understanding was possibly high (low SD of recall similarities and high dISC values). Future studies should examine whether the relationship between dISC and engagement is a function of shared understanding. Furthermore, dFC and BC features could significantly predict the last two phases (i.e., falling and denouement), where the engagement scores were low, but the shared understanding was high. These findings could be considered a first step in exploring the relationship between engagement and shared understanding in dISC, dFC, and BC. However, the results suggest that more investigations should be considered to determine the cognitive processes these features capture.

This is the first study that has characterized the narrative dramatic arc phases at the neural level. Past studies have investigated narrative phases (Bullmore and Sporns, 2009), but have not explored the neural processes that underlie the information processing of each phase. Based on machine-based semantic analysis, Boyd et al. (2020) reviewed ∼40,000 traditional narratives and provided evidence that they consist of a start, which is followed by plot progression, and they then end with a decrease in cognitive tension. They claim that their study was the first that provided empirical support for dramatic arc narrative structures, and they suggested that future studies should focus on exploring psychological aspects induced by this structure. In this study, we used EEG to explore the neuropsychological underpinnings of engagement states for each phase. The results show that dISC, dFC and BC could predict engagement for the rising action, crisis, climax (dISC; Fig. 5), falling action and denouement phase (dFC and BC; Fig. 6*C*,*D*). Importantly, being able to predict engagement in a certain narrative phase does not necessarily requires a high engagement level for the phase. As with the prediction of the whole narrative, the frontal and central electrodes were the most influential. However, the most prominent predictive FCs for the last two phases, which differed from the predictive FCs of the whole narrative, were negative FCs. The last two phases were the least complex in the excerpt used for this study: they only featured one scene with only two characters who had already been introduced, and they did not contain any background action. The higher levels (and low SDs) of the participants’ recall similarity for these phases supported the idea that these phases conveyed more straightforward and less information (Fig. 1*B*). Previous literature on situation models (Zwaan, 1999; Huff et al., 2014; Lin et al., 2019) state that past individual experiences and the information provided by the narrative shape the construction of situation models used for narrative interpretation. Moreover, multiversional thinking proposes the idea that multiple versions of the same narrative can coexist in the audience’s mind as the narrative progresses (Hiskes et al., 2022). We argue that, in our excerpt, the last two phases created much less opportunity for multiversional thinking and complex situation models, which might have been reflected in the pattern of brain activation. This type of brain activation (FC and BC) was able to predict the engagement level, but the activation when facing higher levels of complexity in the narrative (i.e., increased amount of information) could not. dISC, which does not assess connections between regions, could predict engagement levels of the rising action, crisis, and climax phases, which are more complex phases. However, for the exposition phase, none of the three features was able to predict engagement levels. This could be due the characteristics of this phase: it conveys large amounts of information without a particular story line, as it is when the main characters and their relationships, as well as the settings and context are first presented. In summary, out of the five phases of the narrative arc besides the exposition phase, dISC could predict engagement levels of the first three (i.e., rising action, crisis, and climax), and dFC and BC could predict engagement levels of the last two (i.e., falling and denouement). This suggests that the metrics may be sensitive to other factors than only engagement. All of this allows for the exploration of which EEG features and conditions (e.g., higher number of participants, longer periods of stimuli, exposure to several stimuli, type of narrative) could predict phase engagement.

This study contributes to the fields of cognitive narratology and neuroscience by extending our (still limited) knowledge on how brains respond to narratives and how these responses are linked to perceived engagement. In addition, we extended the knowledge on using narratives not only as a means but also as objects of research by analyzing brain responses, perceived engagement, and dramatic arc structure relationships. The movie excerpt selected in this study acted as a fraction of a dramatic arc within the complete narrative (the whole movie). Although the excerpt was perceived as possessing a dramatic arc structure, it did not allow us to make links between the phases and more complex narrative features, such as expectancy and closure. Thus, future studies can advance the study of brain responses to narrative features by presenting a complete narrative to participants. Moreover, given the unique nature of each narrative, there is a need for more studies that use different narratives before our findings can be generalized (Vaccaro et al., 2021). Furthermore, an ambitious step would be investigating how brain responses differ between linear and nonlinear narratives, which opens the door for the exploration of cognitive processes in interactive and immersive narratives (Bruni et al., 2022).

### Limitation and future studies

1. Previous studies have introduced several criteria for calculating intersubject correlation, for example, CCA-based intersubject correlation (Dmochowski et al., 2012). In the current study, a relatively simple method was used to calculate dISC because the main focus was to explore the cognitive processes behind the narrative arc and its phases. However, in the future, other types of intersubject correlation calculation could be used for this purpose.
2. In this study, to calculate the functional connectivity, we used interdependency between electrodes within a single subject as suggested by Song et al. (2021a). However, there is another method called intersubject functional connectivity that is potentially robust to endogenous FC patterns (Simony et al., 2016; Lee et al., 2020), as it calculates interdependency between electrodes of multiple subjects. Future studies are encouraged to use this method and compare the findings.
3. In this study, we used three graph theoretical features (i.e., ND, CC, and BC). However, studies in different fields have used other interesting features to evaluate node centrality and network small worldness (Maffei and Sessa, 2021a, b; Duma et al., 2022). Future studies are encouraged to use these metrics and evaluate how they change within each phase.
4. The video used in this study had narrative phases of various lengths (i.e., shorter length for falling action and denouement phases and longer length for the exposition phase). Further studies are encouraged to choose stimuli with narrative phases of comparable lengths.

## Synthesis

Reviewing Editor: Ifat Levy, Yale University School of Medicine Decisions are customarily a result of the Reviewing Editor and the peer reviewers coming together and discussing their recommendations until a consensus is reached. When revisions are invited, a fact-based synthesis statement explaining their decision and outlining what is needed to prepare a revision will be listed below. The following reviewer(s) agreed to reveal their identity: Antonio Maffei, Buddhika Bellana. Note: If this manuscript was transferred from JNeurosci and a decision was made to accept the manuscript without peer review, a brief statement to this effect will instead be what is listed below.

The manuscript presents results from a very interesting study in which the authors assessed the relationship between cortical activity, measured with EEG, and viewers’ engagement with a short movie excerpt. The main innovation of this work lies in formalizing the narrative structure of the movie excerpt and testing how well cortical activity aligns with this structure.

The authors report several findings: (1) Engagement with a narrative follows the pattern predicted by a narrative arc; (2) Group-level engagement can be predicted from EEG data from a separate sample of subjects (in particular, dynamic inter-subject correlation, dynamic functional connectivity, and graph theoretic betweenness centrality); (3) The ability of neural data to predict engagement is strongest at specific points in the narrative arc.

The manuscript is clearly written and interesting. There are, however, several issues that need to be addressed before the paper can be accepted, as detailed below.

-In the introduction, the authors provide an excellent framework with regards to the formalism of narrative structures, at the expense of a more detailed introduction of the literature on brain processing of audiovisual narratives. For the readership of eNeuro, a more comprehensive introduction to the neural background is important and would put the results in context.

-Relationship between narrative arc and engagement: the authors do not provide much detail about the content of the narrative arc workshop. Were there specific definitions of each phase that were provided to participants? Could those verbatim definitions be provided as supplementary materials? This will clarify how important it is that the self-reported engagement corresponds with the narrative arc (Figure 1A). Is the climax defined (in the context of the workshop) as the most engaging period of the story? If so, why is it so theoretically important (as the authors state throughout the paper) to consider the narrative arc and engagement as distinct entities?

Also, in Figure 1A, what is the Y-axis supposed to be on the narrative arc plot? ‘Expected engagement’ perhaps? Some clarity there, perhaps in the figure caption, would be helpful.

-Phase of arc, engagement, multiversional thinking and shared neural activity: It’s not clear why the authors broke down the dynamic FC and graph analyses into different phases of the narrative arc (Figure 5) but not (as far as reviewers could tell) the dynamic ISC measure.

Conducting the separate phase version of the dynamic ISC analysis would be theoretically informative because the periods in which the EEG data are most predictive of engagement (according to dFC and BC) are not the most engaging periods of the movie (Figure 5).

The authors note that engagement has previously been shown to correspond to neural similarity (page 4, lines 79-84). Here, however, it was in the falling action/denouement when brain activity was most predictive of (group-level) engagement (Figure 5) - and these stages were associated with less engagement than the climax (Figure 1). Furthermore, in the discussion, the authors suggest that the falling action/denouement can be characterized by a lack of ’multiversional thinking’ (i.e., all viewers are more likely to have the same understanding of the story). If neural similarity corresponds to shared understanding (e.g., Nguyen, Vanderwal & Hasson, 2019), one should also see highest dynISC in the falling action/denouement.

If the dynISC measure, indeed, is highest during the falling action/denouement, this would point to an interesting distinction between multiversional thinking and engagement when predicting shared neural activity (i.e., a lack of multiversional thinking matters more for predicting ISC than engagement). However, if dynISC is not highest during the falling action/denouement, more consideration will need to be given to what dynISC is measuring, and how it compares to the other neural predictors (i.e., dFC and BC)

-The authors used 500 microvolts as the threshold for defining a bad channel, which is quite high, even considering that EEG data collected during movie watching are noisier than in typical event-related or resting-state designs. The problem with such a high threshold is that it can detect only channels with really strong contamination, likely leaving bad channels in the data. It is recommended that the authors re-run their analysis using a more conservative threshold like 150 microvolts.

-The authors performed ICA on the data matrix after channel interpolation, which is not advisable. Interpolation introduces linear dependence across the channel, which makes the data matrix rank-deficient. Performing ICA on a rank-deficient matrix yields suboptimal decomposition, which can pose a serious problem, especially if ICs rejection is done automatically. It is recommended that the authors re-run their analysis performing channel interpolation and re-referencing after ICA decomposition and rejection of bad ICs.

-Participants’ responses were resampled to 200 Hz - how was that done? Also, the authors note that resampling was done to match the EEG data, but EEG data were sampled at 1kHz. Were the EEG data subsampled during preprocessing?

-Analysis is focused on raw EEG activity, but we know that the EEG signal is the sum of multiple oscillatory components, which reflects different psychophysiological processes. For instance, in Maffei (2020, https://doi.org/10.1111/psyp.13652), it is shown that intersubject correlation to emotional movies varies as a function of the spectral band considered. Also, Dmochowski (2012) shows that the most correlated component across subjects has a distinctive peak in the alpha band.

In the current form of the results, we do not know which component of the EEG activity is related to the narrative structure and viewers’ engagement.

-The authors define ISC as the simple correlation of raw EEG activity for homologous channels in each pair of participants. This is the simplest approach to computing intersubject correlation, which is directly translated from fMRI research. Nevertheless, the authors must at least acknowledge that there are other, more sophisticated, approaches for quantifying intersubject correlation from EEG data, like the CCA-based approach proposed in Dmochowski et al. (2012) and the spectrally resolved intersubject correlation proposed in Maffei (2020, https://doi.org/10.1111/psyp.13652).

-In the correlation analysis between dISC values and engagement ratings, did the authors correct for multiple comparisons due to testing at multiple cortical sites?

-With regards to the functional connectivity (FC) analysis, why did the authors not use the Inter-Subject Functional connectivity approach introduced in Simony et al. (2016)? Computing functional connectivity within an intersubject approach is better suited to capture the changes in FC driven by the narrative and not reflecting endogenous FC patterns.

-With regards to the use of graph theory metrics applied to dynamic FC, the authors are advised to have a look at the following papers which introduced the multilayer network approach to M-EEG data: Maffei and Sessa (2021, https://doi.org/10.1111/psyp.13786), Maffei and Sessa (2021, https://doi.org/10.1016/j.ynirp.2021.100022), Duma et al. (2022, https://doi.org/10.1016/j.cortex.2022.08.010).

-Direction of prediction between brain and behavior: there should be more discussion of the direction of the predictions. Why is there a negative relationship between dFC and engagement in the falling action and denouement, while dFC and dynISC were associated with a positive relationship with the overall engagement timecourse?

-Theoretical implications of the various neural measures: The authors used a wide array of neural measures to predict engagement, but there was little discussion of the importance of the neural measures relative to one another. Given the relationships between dFC, BC and dynISC (in particular) and engagement, what do these measures tell us about the neural underpinnings of the narrative arc and engagement? The authors are direct about the exploratory nature of this paper, but perhaps it’s worth including an explicit mention of the importance of further considering what we can learn about the neural underpinnings of engagement/narrative arc based on which measures were (and weren’t) predictive.

-The authors should share their analysis code, and are strongly advised to share the data to ensure full reproducibility of their results.

Minor Comments

- It’s not clear why the authors did not statistically test whether the recall similarities were higher for falling action-denouement as compared to the other phases of the narrative arc. They did this for the SD, but it seems that the mean should be higher as well, to support their claim that this period was associated with more shared understanding/similar recall across participants.

- Could the authors discuss why the null distributions look considerably more variable for the falling action/denouement in Figure 5A and B? Could the lack of predictability for the other phases have to do with a lack of variability in the data?

- In the analyses using SVR, the authors are training individual brains on group-level engagement, and then testing whether they can recover group-level engagement from a held-out participant. For dFC, the features were each cell in the 32x32 matrix. For the graph measures, were the features calculated at the level of the electrode (i.e., total number of features = 32)? This was not explicitly stated in the Methods.

- The labels for each ‘part’ of the experiment were not very informative. Particularly, “Neuro part” - perhaps this could be replaced with the more informative label of “EEG dataset”?

- In Figure 4B - why is there an upper and lower triangle? To display negative and positive features? This should be made explicit in the figure caption.

- The term “narrativity” should be defined.

- Why use the terms “dynISC” and “dFC”? Both d and dyn in these cases stand for dynamic. Please consider consolidating to dISC and dFC, unless there’s a reason not to.

- Figure 1A - typo in “denouement”

- Line 496-497: “Furthermore, the results from training and testing the models that were fed the BC features of each phase revealed that, for the dFC feature, the” - is it supposed to be dFC feature here?

- Instead of “fake connectivity” (page 7, lines 163-164) it’s better to use “spurious connectivity”.

- The reference to Song, H., Finn, E. S., & Rosenberg, M. D. is repeated as two different citations.
